# *Boswellia sacra* Extract-Loaded Mesoporous Bioactive Glass Nano Particles: Synthesis and Biological Effects

**DOI:** 10.3390/pharmaceutics14010126

**Published:** 2022-01-05

**Authors:** Kanwal Ilyas, Lamia Singer, Muhammad Asim Akhtar, Christoph P. Bourauel, Aldo R. Boccaccini

**Affiliations:** 1 Department of Materials Science and Engineering, Institute of Biomaterials, University of Erlangen-Nuremberg, Cauerstr. 6, 91058 Erlangen, Germany; kanwal.ilyas@fau.de (K.I.); asim.akhtar@fau.de (M.A.A.); 2 Oral Technology, Dental School, University Hospital Bonn, Welschnonnenstr. 17, 53111 Bonn, Germany; lamia.singer@uni-bonn.de (L.S.); bourauel@uni-bonn.de (C.P.B.)

**Keywords:** bioactive glass, functionalization, *Boswellia sacra*, phytotherapeutics, antibacterial

## Abstract

Bioactive glasses (BGs) are being increasingly considered for numerous biomedical applications. The loading of natural compounds onto BGs to increase the BG biological activity is receiving increasing attention. However, achieving efficient loading of phytotherapeutic compounds onto the surface of bioactive glass is challenging. The present work aimed to prepare novel amino-functionalized mesoporous bioactive glass nanoparticles (MBGNs) loaded with the phytotherapeutic agent *Boswellia sacra* extract. The prepared amino-functionalized MBGNs showed suitable loading capacity and releasing time. MBGNs (nominal composition: 58 wt% SiO_2_, 37 wt% CaO, 5 wt% P_2_O_5_) were prepared by sol-gel-modified co-precipitation method and were successfully surface-modified by using 3-aminopropyltriethoxysilane (APTES). In order to evaluate MBGNs loaded with *Boswellia sacra*, morphological analysis, biological studies, physico-chemical and release studies were performed. The successful functionalization and loading of the natural compound were confirmed with FTIR, zeta-potential measurements and UV-Vis spectroscopy, respectively. Structural and morphological evaluation of MBGNs was done by XRD, SEM and BET analyses, whereas the chemical analysis of the plant extract was done using GC/MS technique. The functionalized MBGNs showed high loading capacity as compared to non-functionalized MBGNs. The release studies revealed that *Boswellia sacra* molecules were released via controlled diffusion and led to antibacterial effects against *S. aureus* (Gram-positive) bacteria. Results of cell culture studies using human osteoblastic-like cells (MG-63) indicated better cell viability of the *Boswellia sacra*-loaded MBGNs as compared to the unloaded MBGNs. Therefore, the strategy of combining the properties of MBGNs with the therapeutic effects of *Boswellia sacra* represents a novel, convenient step towards the development of phytotherapeutic-loaded antibacterial, inorganic materials to improve tissue healing and regeneration.

## 1. Introduction

Bone-tissue engineering is an interdisciplinary field, providing an alternative approach for bone repair and regeneration. Fifty years ago, the first man-made material that bonds strongly to bone was invented, namely silicate bioactive glass (BG) [1]. Bioactive glass dissolves in physiological conditions, releasing ions that stimulate gene-controlling osteogenesis and angiogenesis, making it a very attractive material for bone-regeneration applications [2,3].

Mesoporous bioactive glasses (MBGs) are a special class of BGs exhibiting porosity on the nanoscale, being appealing materials for bone repair and regeneration [4,5,6]. It is proven that MBGs have the ability to support differentiation of osteogenic cells [7,8]. Additionally, the properties of MBGs can be tailored toward specific applications by the functionalization of MBGs with therapeutically active compounds or ions [9,10]. In this context, the synthesis of MBGs in nanoparticulate form, e.g., mesoporous bioactive glass nano particles (MBGNs), is an appealing approach to develop nanocarriers/delivery systems for various biological agents [11]. Based on the approach of using MBGNs as carriers for the release of active compounds and the remarkable efficacy of medicinal plants, MBGNs are being considered as effective candidates for the incorporation of natural herbs and plant extracts aiming to enhance the biological performance and to provide an alternative biomaterial for a variety of applications, including bone regeneration [10].

Plant-derived medical agents are generally known as phytotherapeutic compounds. The use of phytotherapeutic compounds combined with bioactive glasses is at the center of interest of many researchers to provide alternative, synthetic, drug-free biomaterials for a variety of applications, as reviewed elsewhere [10]. Among a number of phytotherapeutic compounds, frankincense (resin of Boswellia species) has been used as an anti-arthritic, anti-inflammatory, analgesic and antibacterial agent [12,13]. Moreover, this compound is commonly used to relieve pain and improve blood circulation [13]. Frankincense is an oleogum resin obtained from the different species of *Boswellia* genus [14]. *Boswellia* resin essential oils contain several pharmacologically active compounds (monoterpenes, sesquiterpenes, ketones) that have antimicrobial properties against both fungi and bacteria, for example, *Escherichia coli, Staphylococcus aureus, Candida albicans* and *Proteus vulgaris* [15,16].

The aim of this study was to prepare extract of frankincense from *Boswellia sacra* and to develop a method to load the prepared extract onto the surface of amino-group-functionalized MBGNs of 58S composition (58 wt% SiO_2_, 37 wt% CaO, 5 wt% P_2_O_5_). Focusing on the functionalization of MBGNs, an effective grafting procedure was developed in order to obtain enhanced biological properties. A release study was carried out to confirm the controlled release of the phytotherapeutic agent. Furthermore, antibacterial properties and the cell-biology response to the phytotherapeutic-laden MBGNs were investigated. 

## 2. Materials and Methods

### 2.1. Synthesis of Mesoporous Bioactive Glass Nanoparticles

Synthesis of mesoporous, bioactive glass nanoparticles (MBGNs) (nominal composition 58S: 58 wt% SiO_2_, 37 wt% CaO, 5 wt% P_2_O_5_) was carried out by sol-gel-modified co-precipitation method. This method of synthesizing 58S MBGNs has been reported previously [17]. Briefly, 21 mM of tetraethyl orthosilicate (TEOS 99% Sigma Aldrich, Darmstadt, Germany) was added into ethanol (99.8% Alfa Aesar, Kandel, Germany), and the pH was adjusted to 2 by adding 1 M nitric acid (HNO_3_). The solution was stirred for 20 min. Separately, 13 mM calcium hydroxide Ca(OH)_2_ (99% Aldrich, Darmstadt, Germany) was dissolved in 40 mL of distilled water and then stirred with the TEOS solution until a homogenous mixture was obtained. This solution was added dropwise in ammonium dibasic phosphate (99% Aldrich, Germany) solution (1.72 mM) with a pH of 11. Finally, the precipitates were collected after 48 h of stirring, filtration was performed to collect the precipitate and washing was subsequently performed with deionized water. MBGNs were dried in the oven at 60 °C. Subsequently, the dried MBGNs were calcined at 680 °C to remove the organic substances and residual nitrate.

### 2.2. Plant Extraction Method

Superior Hojari Frankincense, *Boswellia sacra* gum from Dohfar mountains in Oman, was used (Jeomra Verlag, Georg Huber, Hessen, Germany). The dried gum was frozen overnight to facilitate its grinding process into a powder without sticking to the electric blender. A total of 60 g from the produced resin powder was extracted using a Soxhlet extractor (Carl Roth GmbH + Co. KG, Karlsruhe, Germany) for 4 h with 200 mL ethyl alcohol (70%). Then, the extracted crude gum was removed from the Soxhlet extractor and allowed to soak inside the same alcohol for 4 more days under rotary vibration (CATVM4, Ingenieurbüro CAT, M. Zipperer GmbH, Ballrechte-Dottingen, Germany). The extract was then filtered using Whatman-No. 1 filter paper, and the alcohol was evaporated using a rotary evaporator. The resultant extract was refrigerated at 4 °C in a sealed glass bottle until use.

### 2.3. Surface Functionalization of Mbgns and Loading of Boswellia Sacra Extract

The amino-group functionalization of the MBGN surface was done with (3-Aminopropyl) triethoxysilane (APTES) (Sigma Aldrich, Darmstadt, Germany) [18]. For this purpose, 0.5 g MBGNs was dispersed in 20 vol% APTES solution in acetone for 30 min. The solution was stirred at 70 °C for 6 h under reflux. The functionalized MBGNs were washed three times with deionized water, collected by using a centrifuge and finally dried overnight at 37 °C.

The extract of *Boswellia sacra* for MBGN loading was prepared by adding dried extract into methanol (10% *w*/*v*) and by stirring the solution for 24 h. After that, functionalized MBGNs were added to the solution, and 5 min ultrasonication was performed. Afterwards, the solution was magnetically stirred at 400 rpm at room temperature for one hour. Finally¸ the product was collected by centrifugation at 2000 rpm and dried at room temperature [19]. 

### 2.4. Characterization

#### 2.4.1. Scanning Electron Microscopy 

Field emission scanning electron microscope (FESEM), (Carl Zeiss™ AG, Jena, Germany) was used to evaluate the MBGNs after synthesis and functionalization with *Boswellia sacra* extract. Before analysis, gold sputtering was performed on samples by using a sputter coater (Q150/ S, Quorum Technologies™, Lewes, UK) in order to prevent the charging effect.

#### 2.4.2. Structural and Chemical Analysis

The assessment of the composition of MBGNs was done with the help of energy-dispersive X-ray spectroscopy (EDX) at 20 KV. Moreover, the chemical analysis of the alcoholic extract of *Boswellia sacra* was performed using gas chromatography/mass spectrometry (Agilent Technologies 7890A/Agilent Technologies 7890A) (at the Agriculture Research Centre, Giza, Egypt). The gas chromatography (GC) instrument was equipped with polar Agilent HP-5ms (5%-phenyl methyl polysiloxane) and a capillary column of 30 m in length, 250 μm in diameter and 0.25 μm in thickness. One ml of *Boswellia sacra* extract was diluted in diethyl ether and injected at an injector temperature of 200 °C and detector temperature of 250 °C. Pure helium was used as the carrier gas at a linear velocity of 1 mL/min. Mass spectra were obtained at high-ionization energy of 70 eV (electron Volts), acquisition mass range of 50–800 m/z in positive mode and an interface temperature of 250 °C. The phytochemical compounds present in the test samples were identified and quantified based on comparison of their retention time (min), peak area and spectral-pattern height and mass with the databases of the authentic compounds stored in the National Institute of Standards and Technology (NIST) library and WILEY library, as well as spectral data reported in literature [20]. Furthermore, functional-group analysis was performed by Fourier transform infrared spectroscopy (FTIR) (Shimadzu IRAffinity-1S, Shimadzu Corp, Tokyo, Japan). Spectra were taken in the range of 4000 to 400 cm^−1^ in absorbance mode. X-ray diffraction (XRD) analysis was carried out to evaluate whether MBGNs were amorphous. For this purpose, an X-ray diffractometer (Miniflex 600, Rigaku Corporation, Europe, Neu-Isenburg, Germany) was used. The diffraction patterns were obtained in the 2θ range of 10° to 80° by using Cu Kα radiation.

The pore size, distribution and surface area of MBGNs were evaluated (ASAP2460, Micrometrics Instrument) by observing the nitrogen-adsorption/desorption isotherms and measured with the Barrett–Emmett–Teller (BET) method. Zetasizer Nano ZS instrument (Malvern Instruments, Malvern, Worcestershire, UK) with light-scattering detector positioned at 90° was used to assess the zeta potential of MBGNs. For the measurement, 1 mgmL^−1^ sample concentration was dispersed in deionized water. The analyses were performed in triplicate.

#### 2.4.3. In Vitro Release Studies

The amount of adsorbed *Boswellia sacra* was evaluated by using UV-Vis spectroscopy at 215 nm wavelength. For this purpose, 50 mg of *Boswellia sacra*-loaded MBGNs was compressed into 6 mm molds, and then the obtained pallets were immersed in 10 mL phosphate buffer solution (PBS) in an incubator at 37 °C for 2, 4, 6, 24, 48, 72, 96, 120, 144 and 168 h. After each time point, 2 mL solution was taken out and replaced with fresh 2 mL of PBS. The loading capacity of MBGNs and functionalized MBGNs was evaluated by measuring the absorbance *of Boswellia sacra* solution before and after the addition of MBGNs. The release study was done in triplicate.

#### 2.4.4. Antibacterial Characterization

Two classic pathogenic bacteria, *Staphylococcus aureus* (*S. aureus*) and *Escherichia coli* (*E. coli*), were used to assess the antibacterial activity of MBGNs before and after loading with *Boswellia sacra* extract. The evaluation of the antimicrobial activity was done by an agar diffusion test. Both bacteria were grown at 37 °C for 24 h in lysogeny broth medium (Luria/Miller) (LB Medium) (Carl Roth GmbH, Karlsruhe, Germany), a medium for bacterial culture. Samples were prepared in a 6 mm diameter disc by compacting MBGNs in a mold. The disc diffusion method was used for the antibacterial evaluation of MBGNs, functionalized MBGNs and *Boswellia sacra*-loaded MBGNs. Three discs of MBGNs from each group were placed on the surface of lysogeny broth agar (Luria/Miller) (LB Agar) (Carl Roth GmbH, Karlsruhe, Germany) plates that had been seeded previously with the tested strain of bacteria. A well diffusion method was used for the antibacterial assessment of the *Boswellia sacra* extract. Wells with a diameter of 4 mm were created. After spreading the tested strain of bacteria, the wells were filled with the *Boswellia sacra* extract. The agar plates were then placed in an incubator at 37 °C for 24 h. The inhibition of growth was confirmed by observing the presence of a clear zone around the samples. This zone was measured to indicate the degree of inhibition against the bacterial species.

#### 2.4.5. In Vitro Cytocompatibility

Human osteoblast-like cells (MG-63) (Sigma Aldrich, Germany) were used to evaluate the in vitro cytocompatibility of the samples. Cells were cultured in Dulbecco’s Modified Eagle Medium (DMEM, Gibco, Thermo Fisher Scientific, Schwerte, Germany) at 37 °C in an incubator. The cytotoxicity of as-synthesized MBGNs and *Boswellia sacra*-loaded MBGNs was assessed by following the standard elution test protocol (ISO 10993–5) [21]. To obtain the extracts, as-synthesized MBGNs and *Boswellia sacra*-loaded MBGNs were incubated in DMEM. A concentration of 0.1 mg mL^−1^ was used for both samples and incubated in falcon tubes for 24 h at 37 °C. In the meantime, 5 × 10^4^ cells were seeded in a 24-well plate. After the given time, samples were centrifuged, and extracts were collected. The obtained eluate was placed in a pre-cultured cell monolayer without any solid particles and jointly incubated for 24 h and 72 h in a cell culture incubator at 37 °C. Subsequently, medium from all wells of the well plate was removed and cells were then thoroughly washed with PBS (phosphate-buffered saline). Cells in untreated condition were used as control. Mitochondrial activity of the cells was evaluated by using the WST-8 method “2-(2-methoxy-4-nitrophenyl)-3-(4-nitrophenyl)-5-(2,4-disulfophenyl)-2H-tetrazolium” (CCK-8 Kit, Sigma Aldrich, Germany). The reaction product was measured at 450 nm after 4 h of incubation at 37 °C. Relative cell viability was calculated according to the equation:

(1) $$Relative\;viability\;of\;cells\;\left(\%\right)=\frac{OD_{sample}-OD_{blank}}{OD_{reference}-OD_{blank}}\times 100$$

where *OD_sample_* is the optical density of cells grown in cell culture medium containing the eluate, *OD_blank_* is the optical density of WST reactant and *OD_reference_* is the optical density of cells grown in cell culture medium with no eluate.

The qualitative assessment of cell viability and morphology was done via live staining with Calcein AM (Life Technologies, Darmstadt, Germany) and DAPI (4′,6-diamidino-2-phenylindole). The process of cell staining was according to the supplier’s manuals. Once the staining was done, a fluorescence microscope (Axio Scope A1, Carl Zeiss Microimaging GmbH, Jena, Germany) was used to obtain fluorescence images. 

### 2.5. Statistical Analysis

The experiments were performed in triplicate; all results are represented as mean and standard deviations (SDs). The statistical significance of biological studies was evaluated by using one-way ANOVA test with *p* < 0.05 (*) considered statistically significant. 

## 3. Results and Discussion

### 3.1. Morphological Analysis

**Figure 1:**
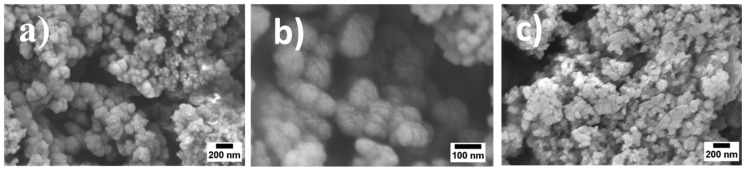
SEM images showing surface morphology of MBGNs at two magnifications (**a**,**b**) and *Boswellia sacra*-loaded MBGNs (**c**).

SEM micrographs of MBGNs and *Boswellia sacra*-loaded MBGNs are presented in Figure 1. It is evident from SEM observations that MBGNs are agglomerated. Moreover, the prepared MBGNs have a heterogeneous particle size, which turned the MBGNs into a multilevel pore structure. The controlled aggregation model explains the production of nanoparticles with spherical agglomerates and heterogeneous particle sizes [22,23]. According to this model, the formation of silicate particles involves two stages: nucleation and growth. The first stage (nucleation of silicate particles) occurs during the entire course of reaction. Initially, primary particles or small nuclei are formed. After that, dimer, trimer and ultimately larger particles or secondary particles are formed by combination of primary particles via condensation reaction. The formation of spherical particles depends on the reaction conditions, such as the pH of the medium. It was observed in previous studies that the pH of the medium is capable of modulating the hydrolysis and condensation reactions [24]. In this study, the addition of ammonia solution to the acid-catalyzed sol increased the pH of the reaction medium to greater values than the isoelectric point of silicate particles [24]. Therefore, the increased rate of condensation reactions caused a decrease in gelation time. Consequently, a controlled rate of aggregation of primary particles resulted in the production of MBGNs [23]. Moreover, Figure 1 shows SEM images of *Boswellia sacra*-loaded MBGNs. It can be observed that the morphology of MBGNs changed after loading the *Boswellia sacra* extract. This change in morphology is likely due to the surface-layer formation after loading the functionalized MBGNs [25].

### 3.2. Chemical Analysis

**Figure 2:**
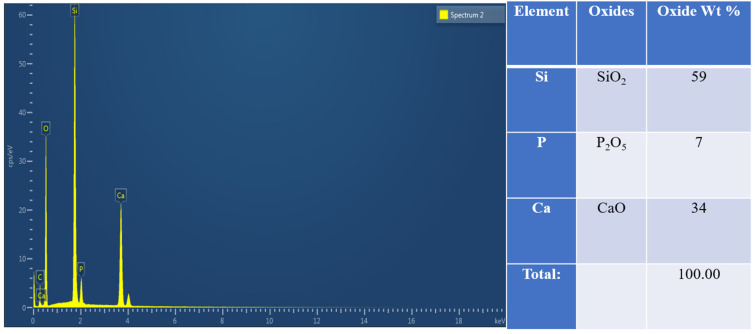
EDX analysis describing the weight percentages of oxides in MBGNs.

The EDX spectra of as-synthesized MBGNs are shown in Figure 2, which proves the presence of Si, Ca and P in the nanoparticles. The chemical composition (wt%) was estimated based on the atomic ratio obtained and is listed in the table shown in Figure 2. The comparison of EDX values with the theoretical values of the MBGN composition indicates the successful synthesis of 58S MBGNs. 

The chemical characterization of the phytochemical compounds of *Boswellia sacra* extract was performed using a combination of two analytical techniques: gas chromatography/mass spectrometry (GC/MS). Gas chromatography has the ability to separate compounds with high resolution, while mass spectrometry can accurately identify and quantify them. Chemical analysis revealed the presence of 43 volatile and semi-volatile active compounds (Table 1). Terpenes were detected in considerable amounts in the extract, including hydrocarbons (α-pinene, β- pinene (Myrtenol), limonene, p-cymene), monoterpene alcohols (linalool, α-terpineol), phenols (7-Hydroxychromanone) and sesquiterpene (Himbaccol, α-Selinene, α-Cadinol, Juniperol). Terpenes and their derivatives are known for their satisfactory antimicrobial activities [26]. Coumarins (Dimethyl-4-hydroxycoumarin, 3-(3,4-Dimethoxyphenyl)-7-hydroxy-4-phenylcoumarin, 7-Methoxy-3-(4-methoxyphenyl) coumarin, 5,7-Dihydroxy-4-methylcoumarin) have profound antibacterial activity against both vulnerable and resistant pathogens by damaging the bacterial cell membrane, causing denaturation of proteins and affecting cell-membrane permeability [27]. Saponins (Squalene, Kaur-16- ene) cause alteration of the bacterial cell wall and leakage of proteins and certain enzymes [28], whereas, flavonoids (2′- Hydroxy-2,4,4′,5-tetramethoxychalcone, Quercetin 3′-methyl ether, Isovitexin, Tetramethoxyflavanone, Trimethoxyflavone and Apigenin 8-Cglucoside) trigger inhibition of nucleic-acid synthesis and cytoplasmic-membrane dysfunction [29].

### 3.3. FTIR

**Figure 3:**
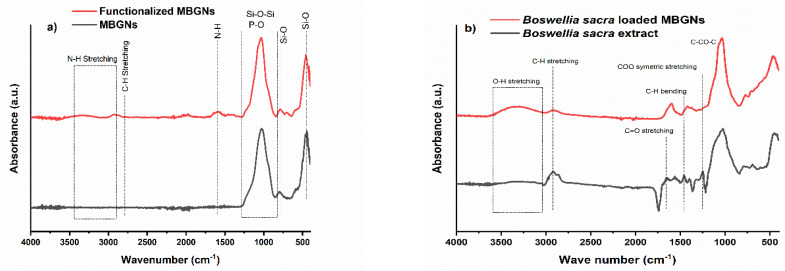
FTIR spectra of MBGNs and functionalized MBGNs (**a**), *Boswellia sacra* extract and *Boswellia sacra*-extract-loaded MBGNs (**b**). The indicated relevant peaks are discussed in the text.

MBGN functionalization by APTES was confirmed by using FTIR and recording spectra before and after functionalization. The FTIR spectra are shown in Figure 3a. A peak at 1029 cm^−1^ can be seen, which is mainly due to the Si-O-Si stretching vibrations. The band at 801 cm^−1^ represents the Si-O symmetric stretching vibration in MBGNs [30], and the Si-O bending vibration can be detected from the presence of a peak at 447 cm^−1^. After functionalization, the additional peaks can be seen at 698, 1589 and 2928 cm^−1^, confirming the APTES modification of MBGNs [31]. These peaks are associated to deformation-mode NH_2_ groups and the stretching mode of C-H bonds. Therefore, these peaks confirm the functionalization of MBGNs by the adsorption of APTES onto the surface of the glass particles [30]. The symmetric and asymmetric stretching bands of Si-O-Si for physiosorbed APTES appeared at 1035 cm^−1^ and 1081 cm^−1^, respectively [30]. Besides, the broad band at 3431 cm^−1^ is ascribed to the N-H stretching that overlapped with the stretching peak of the O-H group [30]. 

FTIR spectra of dried extract of *Boswellia sacra* and *Boswellia sacra*-loaded MBGNs are shown in Figure 3b. The FTIR spectra of *Boswellia sacra* showed peaks at 2917 cm^−1^ and 1662 cm^−1^, corresponding to C-H stretching and C=O stretching of aryl acid [32], respectively. Moreover, spectra showed the characteristic peaks of carboxylate COO symmetric stretching at 1388 cm^−1^, stretching at 1252 cm^−1^ related to the C-CO-C in aryl ketone. Moreover, the 1026 cm^−1^ peak is associated to the ring structures of cyclohexane [32]. It can be observed that after loading *Boswellia sacra* extract onto MBGNs, some of the characteristic peaks of *Boswellia sacra* disappeared or changed their position and intensity, which indicates that the plant extract established an interaction with the bioactive glass surface.

### 3.4. XRD Analysis

**Figure 4:**
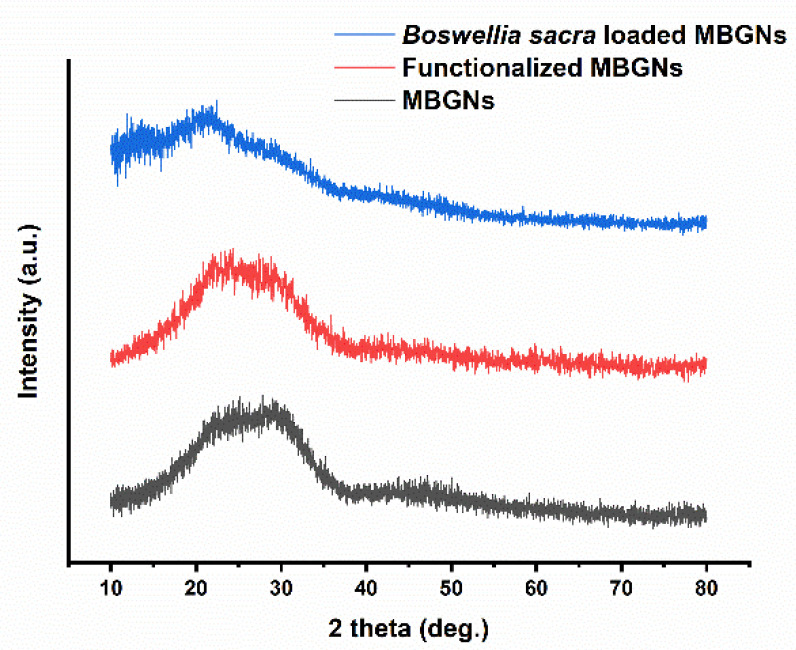
XRD patterns of MBGNs, functionalized MBGNs and after loading of *Boswellia sacra* extract.

The XRD patterns of MBGNs before and after functionalization and of *Boswellia sacra*-loaded MBGNs are shown in Figure 4. In order to confirm whether the synthesized MBGNs and *Boswellia sacra*-loaded MBGNs are amorphous, samples were characterized with XRD. The XRD pattern of MBGNs shows no sharp diffraction peaks, which demonstrates that the MBGNs have an amorphous structure (broad band at 2θ~28° represents typical amorphous characteristics of a glass). Moreover, functionalized MBGNs and *Boswellia sacra*-loaded MBGNs exhibit no crystalline diffraction peaks. This demonstrates that the functionalization of MBGNs and loading of *Boswellia sacra* extract did not have any effect on the amorphous structure of MBGNs.

### 3.5. Textural Properties 

**Figure 5:**
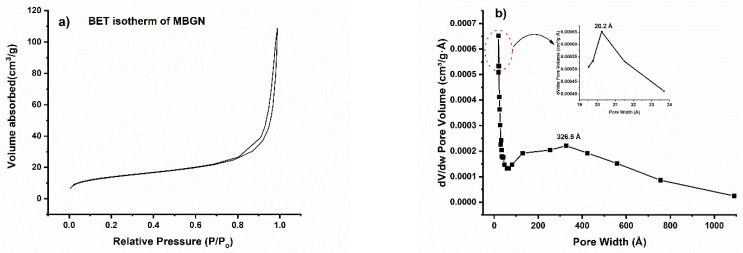

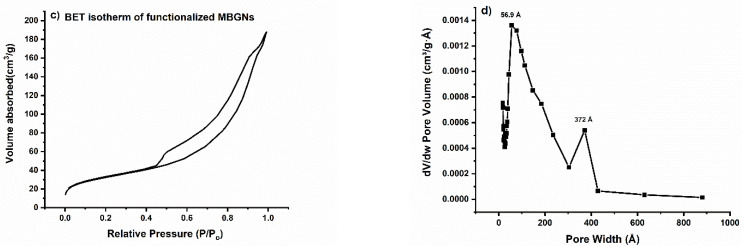
Nitrogen-adsorption/desorption isotherms of prepared MBGNs (**a**), pore-size distribution (**b**), nitrogen-adsorption/desorption isotherms of prepared functionalized MBGNs (**c**) and pore-size distribution (**d**).

From the BET analysis of prepared MBGNs, the nitrogen-adsorption/desorption isotherm was obtained that demonstrated IUPAC type IV isotherm, indicating that the synthesized MBGNs are mesoporous. The isotherm showed the characteristic hysteresis loop. The loop had a different path for the desorption and adsorption branches at relatively high P/P_0_ values. According to IUPAC classification, an isotherm with that kind of hysteresis loop signifies that the material has pores in the mesoporous range (2 nm–50 nm) [23]. From the adsorption isotherm, the pore size distribution was measured by employing the BJH method for MBGNs, and results are presented in Figure 5. It can be noticed that the pore-size distribution of the prepared MBGNs is in the mesoporous range. Moreover, the pore-size distribution for the MBGNs showed multimodal distribution. For example, the highest mode was in the mesoporous range (20.2 Å), while the other mode was less prominent but had a larger pore size (326.5 Å). In Table 2, the textural properties are listed, indicating that MBGNs and functionalized MBGNs are in the mesoporous range. However, after functionalization, MBGNs showed a marked mesoporous character, including larger pore size, as well as higher surface area and pore volume. As APTES is a coupling agent with amino groups that react with OH groups on the surface of BG particles, a change in the mesoporous characteristics appears after functionalization [33]. According to Zhang et al., collision of molecules during the grafting process may also cause the change in pore size [34].

### 3.6. Zeta Potential

The measurement of zeta potential (see Table 3) is a convenient approach to determine the presence of silane molecules on the surface of BGs. Furthermore, glass-surface modification or change in glass-surface charge can be effectively identified by using this technique [25]. It was observed that the surface of the MBGNs present a negative zeta potential value (−21.7 mV). Therefore, to load the negatively charged organic compounds onto the surface of MBGNs was a challenge due to the intrinsic repulsion caused by the compounds [35]. In order to alter the charge of the surface of MBGNs, amino groups should be grafted. The amino group had a nitrogen atom with lone-pair electrons that could be combined with the proton from water to produce a positively charged NH^3+^. The surface of the MBGNs was grafted with amino groups after surface modification, which was confirmed by the FTIR evaluation (Figure 3). As result of the functionalization, the zeta potential of the MBGNs became less negative (−11.8 mV), which confirms the functionalization of MBGNs with NH^3+^. This functionalization strategy should enable loading of negatively charged drugs or other organic compounds [31]. On the other hand, *Boswellia sacra*-loaded MBGNs showed a more negative zeta potential value (−32.2 mV). According to Kaasalainen et al. [36], this may be because of termination of hydrocarbon chains with a –COOH functional group onto the surface of the molecules (*Boswellia sacra*). This effect turns the zeta potential to a more negative value.

### 3.7. In Vitro Release Studies

**Figure 6:**
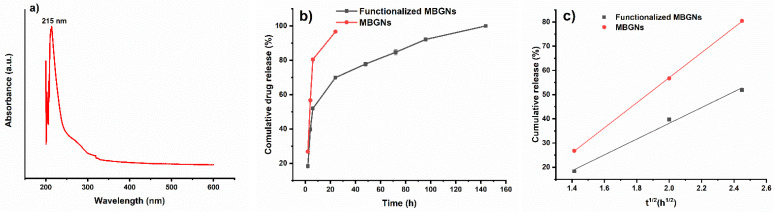
Maximum absorbance (λmax) of *Boswellia* *sacra* extract (**a**), in vitro release study of *Boswellia sacra*-loaded MBGNs and functionalized MBGNs in PBS (**b**) and data for the first 6 h fitted to the Higuchi model (**c**).

Maximum absorbance (λ_max_) of *Boswellia sacra* extract was observed at a wavelength of 215 nm. By using this λ_max_, a calibration curve was plotted by using solutions with known concentration of *Boswellia sacra* in order to find the unknown concentration for the release study. A release study for MBGNs before and after functionalization was carried out in order to evaluate the effect of functionalization on drug loading and its release. The *Boswellia sacra* release profile is shown in Figure 6b. The release study was done at 2, 4, 6, 24, 48, 72, 96, 120, 144 and 168 h. An initial burst release was observed in the case of functionalized *Boswellia sacra*-loaded MBGNs (about 52% in 6 h), followed by a constant and slower release up to 168 h. The initial burst release indicates that some *Boswellia sacra* molecules that were adhered onto the surface of the MBGNs diffused out rapidly into the solution [31]. This might be the main cause of the faster release of *Boswellia sacra* molecules in the early stage. After that, a controlled release of extract from MBGNs was measured. The reason for the controlled release might be that most of the *Boswellia sacra* molecules adsorbed onto the surface of the MBGNs via electrostatic interactions or were accumulated in the mesoporous channels [31]. However, the cumulative release from MBGNs reached 99.9% of the total loading after 144 h. On the other hand, in case of non-functionalized MBGNs, 96.6% of the drug was released within 24 h. It has been observed in previous studies that a significant amount of drug can be released from MBGNs within 24 h [37]. Jiang et al. [31], for example, observed that after amino functionalization, the drug-loading capacity, as well as drug release time, of MBGNs increased significantly. It was also seen in the current study that the loading capacity on functionalized MBGNs was higher (9.9 ± 0.1 mg) than that on MBGNs (1.9 ± 0.2 mg) (on 500 mg MBGNs). The reason might be the change in the surface charge after functionalization. Thus, the modified MBGNs were able to load a higher concentration of drug molecules [31,38].

Furthermore, the release of *Boswellia sacra* from mesoporous channels was controlled by Fickian diffusion. The first 6 h were chosen to assess the *Boswellia sacra* release kinetic from MBGNs (functionalized and non-functionalized), for which the data were fitted by using the Higuchi model [31,38]:

Q = kt^1/2^

where Q is the amount of extract released from the MBGNs in time, t, and k is the Higuchi dissolution constant. According to Figure 6c and Table 4, the cumulative amount of released *Boswellia sacra* is linearly proportional to the square root of time, which indicates that the drug release is governed by a diffusion mechanism. Thus, the initial release of *Boswellia sacra* from both the MBGNs and functionalized MBGNs can be effectively controlled and predicted.

### 3.8. Antibacterial Studies

**Figure 7:**
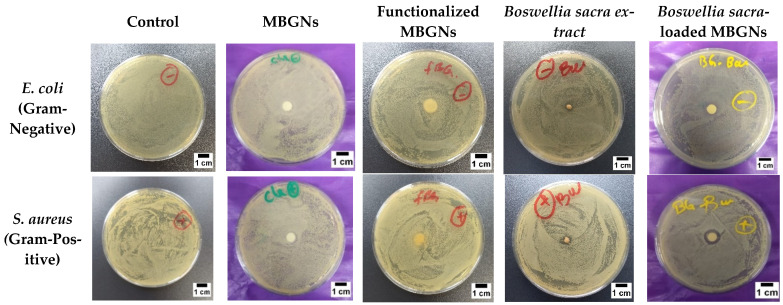
Optical images of agar plates after 24 h of incubation for different studied samples.

The antibacterial behaviour of MBGNs, functionalized MBGNs and *Boswellia sacra*-loaded MBGNs was evaluated with the method of zone-of-inhibition formation. The results of antibacterial studies against Gram-positive (*S. aureus*) and Gram-negative (*E. coli*) bacteria after 24 h of incubation are shown in Figure 7. Results revealed that MBGNs and functionalized MBGNs did not show a significant zone of inhibition. However, after loading MBGNs with plant extract, the antibacterial properties were significantly enhanced. Antibacterial studies showed that *Boswellia sacra*-loaded MBGNs have a significant antibacterial effect against *S. aureus* (Gram-positive) bacteria. The zone of inhibition is clearly visible in the case of *S. aureus*. However, a slight depletion of Gram-negative (*E. coli*) bacterial colonies was also observed. Several studies have been reported regarding the antibacterial activity of extracts from different species of *Boswellia sacra*, and it is proven that, as compared to Gram-negative bacteria, extracts exhibit better antibacterial activity against Gram-positive bacteria [39,40,41].

The antibacterial activity of *Boswellia sacra*-loaded MBGNs could be explained on the basis that it possesses different biologically active constituents, including monoterpenes (α-pinene, limonene), sesquiterpenes and pentacyclic triterpene (alpha-boswellic acid and beta-boswellic acid). The antibacterial activity of these terpenoids could be attributed to cell-membrane disruption and the increase in cell-membrane permeability causing the release of vital intracellular constituents [42]. Moreover, the detected coumarins, saponins and flavonoids might have played an important role in the enhancement of the antibacterial activity through their ability to damage the bacterial cell wall and inhibit nucleic-acid synthesis. [27,28,29]. 

Conversely, the lower antibacterial activity of the *Boswellia sacra*-loaded MBGNs against Gram-negative bacteria can be explained by the presence of the external lipophilic membrane of *E. coli*. This outer layer is mainly composed of lipopolysaccharide molecules that form a hydrophilic permeability blockade, which protects against the effects of highly hydrophobic compounds [41].

### 3.9. Cytotoxicity Analysis

**Figure 8:**
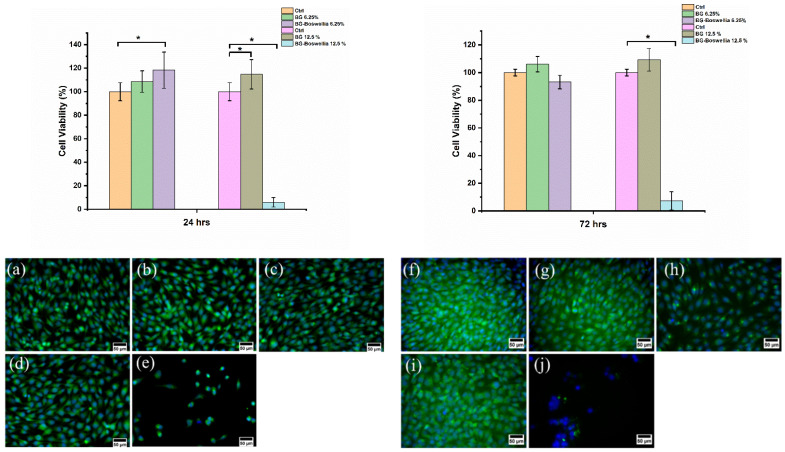
Graphs representing the time- and concentration-dependent effect of *Boswellia sacra*-loaded MBGNs on the MG-63 cell line and morphological responses of cells exposed to different concentrations after 24 h, presented in Ctrl (**a**), MBGNs 6.25% (**b**), MBGNs drug 6.25% (**c**), MBGNs 12.5% (**d**), MBGNs drug 12.5% (**e**), and after 72 h are presented in Ctrl (**f**), MBGNs 6.25% (**g**), MBGNs drug 6.25% (**h**), MBGNs 12.5% (**i**) and MBGNs drug 12.5% (**j**). Significant differences in cell viability values are marked by an asterisk (*).

An indirect cytotoxicity assessment was carried out according to ISO 10993-5 standard. In these tests, 0.1 g of each sample in 1 mL culture medium was used. The eluate was diluted, and two concentrations (12.5% and 6.25%) were used, which gave rise to MBGN concentrations of 12.5 mg/mL and 6.25 mg/mL, respectively. Cell viability was assessed after 24 h and 72 h. It was observed that a lower concentration (6.25%) of eluate from *Boswellia sacra*-loaded MBGNs significantly increased cell viability after 24 h of incubation. However, no significant change in cell viability was observed from the same concentration of eluate from non-loaded MBGNs (Figure 8). It has been reported that boswellic acid, which is the main component of the *Boswellia sacra* extract, might stimulate osteoblast cell differentiation and be capable of inhibiting osteoclastogenesis by suppressing NF-κB and TNF-α signaling [43,44]. However, a higher concentration of eluate (12.5%) shows cytotoxic behavior of drug-loaded MBGNs. This result confirms the concentration-dependent cytotoxic behavior of the drug. Interestingly, the higher concentration of eluate from MBGNs enhanced cell viability after 24 h of incubation (Figure 8). There are numerous examples that prove the ability of bioactive glasses to boost osteoblast activity considering the release of Ca and Si ions [2,3,11]. Furthermore, the lower concentration (6.25%) of drug-loaded MBGN eluate did not show cytotoxic effects, even after 72 h of culture. In conclusion, it can be stated that the cytotoxic behavior of drug-loaded MBGNs is concentration-dependent. A possible synergistic effect of *Boswellia sacra* extract and ions released from MBGNs remains an interesting subject for future research.

## 4. Conclusions

This work developed a facile approach to load the phytotherapeutic agent *Boswellia sacra* onto surface-modified mesoporous bioactive glass nanoparticles. Surface functionalization of MBGNs with amino groups was performed successfully by using APTES and was confirmed by FTIR and zeta-potential analysis, which showed NH group attachment onto the bioactive glass surface. The phytotherapeutic agent *Boswellia sacra* was successfully loaded onto the amino-group-modified MBGNs. SEM micrographs showed that after loading *Boswellia sacra*, the morphology of MBGNs changed; a layer was formed on the surface of MBGNs, which confirmed the loading of the phytotherapeutic agent. Antibacterial studies revealed that drug-loaded MBGNs exhibited antibacterial properties against Gram-positive (*S. aureus*) bacteria. A slight depletion of Gram-negative (*E. coli*) bacterial colonies was also observed around *Boswellia sacra*-loaded MBGNs. *Boswellia sacra* has strong antibacterial properties against Gram-positive bacteria. The drug-release study indicated a burst release (70% of drug) at 24 h. After that, a sustained release of the drug was observed. MG-63 cell viability using two different eluate concentrations was assessed after 24 h and 72 h. It was observed that the high-concentration (12.5%) eluate gave rise to a cytotoxic effect, even after 24 h. However, a lower-concentration eluate (6.25%) showed cell viability until 72 h. The present study suggests that amino-group-functionalized mesoporous bioactive glass nanoparticles loaded with phytotherapeutic agents represent a versatile combination for antibacterial and tissue-engineering applications. 

## Figures and Tables

**Table 1:** Results of the gas chromatography–mass spectrometry (GC/MS) analysis of *Boswellia Sacra*.

	Retention Time (Min)	Compounds	% Area
1	4.633	α-Pinene	36.96
2	5.092	7-Methoxy-3-(4-methoxyphenyl)coumarin	1.81
3	5.724	Limonene	4.92
4	6.741	Myrtenol	0.45
5	6.999	5,7-Dihydroxy-4-methylcoumarin	1.34
6	7.364	α-Thujenal	0.51
7	7.581	p-Cymen-7-ol	0.89
8	8.184	Bornyl acetate	0.41
9	8.93	α-Terpineol	0.37
10	9.25	α-Selinene	0.42
11	9.406	δ-Guaiene	1.39
12	9.673	Caryophyllene	0.51
13	9.964	Humulene	0.49
14	10.099	Longifolene	0.47
15	10.23	γ-Gurjunene	1.59
16	10.468	Epicubebol	0.66
17	10.956	cis-Sesquisabinene hydrate	0.53
18	11.161	Farnesol	0.67
19	11.342	Himbaccol	0.66
20	11.506	α-Cadinol	0.97
21	11.891	(-)-Spathulenol	0.48
22	12.621	Thunbergene	0.41
23	13.441	β-Santalol	0.50
24	13.667	Lanceol, cis	0.52
25	13.691	β-Elemen	0.77
26	13.888	α-Terpinyl acetate	0.67
27	13.966	3,6,3′,4′-Tetrahydroxyflavone	0.63
28	14.249	β Carotene	3.12
29	14.512	Kaur-16-ene	0.97
30	14.815	Squalene	3.66
31	14.922	Ledol	13,22
32	15.11	7,3′,4′,5′-Tetramethoxyflavanone	0.59
33	15.398	Quercetin 3′-methyl ether	0.52
34	16.414	Ledene	12.46
35	17.657	Apigenin 8-C-glucoside	0.42
36	18.006	2′-Hydroxy-2,4,4′,5-tetramethoxychalcone	0.48
37	18.309	Juniperol	0.68
38	18.752	Isovitexin	0.51
39	19.814	6,2′,3′-Trimethoxyflavone	0.54
40	20.118	(-)-Globulol	0.50
41	21.315	3-(3,4-Dimethoxyphenyl)-7-hydroxy-4-phenylcoumarin	0.83
42	22.546	7-Hydroxychromanone	0.75
43	22.878	4-Hydroxy-7-methoxy-3-(4-methoxyphenyl)coumarin	0.77

**Table 2:** Textural properties of the MBGNs developed according to the results of the BET analysis.

Type of BG	Pore Size (nm)	Pore Volume (cm^3^ g^−1^)	BET Surface Area (m^2^/g)
MBGNs	2.02	0.2	100.5
Functionalized MBGNs	5.69	0.29	111.2

**Table 3:** Zeta potential of MBGNs before and after loading of *Boswellia sacra* extract.

Samples	Zeta Potential (mV)	Std. Dev.
MBGNs	−21.7	±0.8
Functionalized MBGNs	−11.8	±0.2
*Boswellia sacra*-loaded MBGNs	−32.2	±1.0

**Table 4:** The Higuchi dissolution constant and regression factor of MBGNs and functionalized MBGNs.

Equation	Q = Kt^(1/2)^	
Functionalized MBGNs	K	32.61819
	R^2^	0.99347
MBGNs	K	51.77643
	R^2^	0.99989

## Data Availability

The data presented in this study are available on request from the corresponding author.
